# The Role of Neuroglial Metabotropic Glutamate Receptors in Alzheimer’s Disease

**DOI:** 10.2174/1570159X19666210916102638

**Published:** 2023-02-01

**Authors:** Khaled S. Abd-Elrahman, Shaarika Sarasija, Stephen S.G. Ferguson

**Affiliations:** 1 Department of Cellular and Molecular Medicine, University of Ottawa 451 Smyth Road, Ottawa K1H 8M5, Ontario, Canada;; 2 Department of Pharmacology and Toxicology, Faculty of Pharmacy, Alexandria University, Alexandria 21521, Egypt

**Keywords:** mGluR, GPCR, tau, amyloid beta, neurodegeneration, astrocytes, microglia, oligodendrocytes

## Abstract

Glutamate, the major excitatory neurotransmitter in the brain exerts its effects *via* both ionotropic glutamate receptors and metabotropic glutamate receptors (mGluRs). There are three subgroups of mGluRs, pre-synaptic Group II and Group III mGluRs and post-synaptic Group I mGluRs. mGluRs are ubiquitously expressed in the brain and their activation is poised upstream of a myriad of signaling pathways, resulting in their implication in the pathogenesis of various neurodegenerative diseases including, Alzheimer’s Disease (AD). While the exact mechanism of AD etiology remains elusive, β-amyloid (Aβ) plaques and hyperphosphorylated tau tangles remain the histopathological hallmarks of AD. Though less electrically excitable, neuroglia are a major non-neuronal cell type in the brain and are composed of astrocytes, microglia, and oligodendrocytes. Astrocytes, microglia, and oligodendrocytes provide structural and metabolic support, active immune defence, and axonal support and sheathing, respectively. Interestingly, Aβ and hyperphosphorylated tau are known to disrupt the neuroglial homeostasis in the brain, pushing them towards a more neurotoxic state. In this review, we discuss what is currently known regarding the expression patterns of various mGluRs in neuroglia and how Aβ and tau alter the normal mGluR function in the neuroglia and contribute to the pathophysiology of AD.

## 1 INTRODUCTION

Glutamate is the major excitatory neurotransmitter in the mammalian central nervous system (CNS) and it plays a pivotal role in synaptic plasticity and memory consolidation. Glutamate activates two classes of glutamate receptors in the brain: ionotropic and metabotropic. Inotropic glutamate receptors are ligand-gated ion channels responsible for the fast excitatory signaling of glutamate and include N-Methyl-d-Aspartate (NMDA), α-Amino-3-hydroxy-5-methyl-4-isoxa-zolepropionic Acid (AMPA) and kainate receptors. Metabotropic glutamate receptors (mGluRs) belong to the G protein-coupled receptor (GPCR) superfamily, the most abundant cell surface receptor family in the human genome and they mediate the slow excitatory signaling of glutamate primarily *via* G protein-regulated second messenger pathways [1-4]. mGluRs form both constitutive homo- and hetero-dimers and belong to the class C subfamily of GPCR that are characterized by a large extracellular N-terminus that forms venus flytrap-like domain [5-7]. There are eight identified mGluR subtypes that are ubiquitously expressed throughout the brain and are divided based on sequence homology,pharmacology, and signal transduction pathways into three subgroups, Group I (mGluR1 and mGluR5), Group II (mGluR2 and mGluR3), and Group III (mGluR4/6-8) [1-3, 8]. Group I mGluR are located mostly post-synaptically surrounding ionotropic receptors to modulate neuronal excitability [9, 10]. Group II and III mGluRs are localized primarily pre-synaptically and act as autoreceptors to inhibit glutamate release [2, 11, 12]. mGluRs are ubiquitously expressed in the brain and along with their neuronal localization, various mGluR subtypes are also detected in neuroglia [13, 14]. Due to its central role in neuronal signaling, tight regulation of extracellular concentration of glutamate is critical. Abnormally high extracellular glutamate levels can lead to neuronal dysfunction and neurodegeneration as a result of excitotoxicity; a condition brought on by excessive glutamatergic stimulation that causes spikes in intracellular calcium levels and triggers neurotoxic signaling events [13, 15, 16]. In this review, we will focus on the functional role of mGluRs in brain neuroglia and discuss their potential contribution to Alzheimer’s Disease (AD) pathophysiology.

## 2 mGluR SIGNALING MECHANISMS

Like all GPCRs, the activation of mGluRs triggers a conformational change in the receptor that initiates the exchange of guanosine diphosphate (GDP) for guanosine triphosphate (GTP) on the α-subunit of the heterotrimeric (α, β and γ subunits) G-protein complex [2, 6, 17]. Receptor signal transduction is usually mediated by the dissociative activation of functional α and βγ G protein subunits that serve to modulate the function of multiple and diverse effector proteins, including enzymes, ion channels and transcription factors. Group I mGluRs preferentially couples to Gα_q/11_ proteins that activate phospholipase β1 (PLCβ1) to hydrolyze membrane phospholipids and generate diacylglycerol (DAG) and inositol 1,4,5 trisphosphate (IP_3_). The latter activates IP_3_ receptors (IP_3_R) on the endoplasmic reticulum to trigger the release of intracellular Ca^2+^ resulting in a rise in the cytosolic Ca^2+^ levels and increased synaptic excitability. On the other hand, DAG remains attached to plasma membrane and, either together with released Ca^2+^ or on its own, activates multiple downstream protein kinase C (PKC) isoforms that can then modulate the functional activity of a variety of other kinases, receptors, and ion channels that are important for neuronal function and synaptic plasticity [1, 10, 18, 19]. Additionally, Group I mGluRs modulate extracellular Ca^2+^ entry and Ca^2+^-dependent signaling required for synaptic function *via* its physical interaction with NMDARs and the activation of Src family Fyn kinase that can alter NMDAR activity [20-22]. Group I mGluRs also activate extracellular signal-regulated kinase (ERK) and phosphoinositide 3-kinase (PI3K)/ mammalian Target of Rapamycin (mTOR) to promote rapid synaptic protein translation required to support plasticity changes associated with formation of memory [23-27]. Group I mGluRs also regulate the autophagic clearance of misfolded proteins and toxic cargos, a function that is essential to preserve neuronal integrity [28-31].

Both Group II and Group III receptors share a significant sequence homology (70%), and normally inhibit glutamatergic neurotransmission. Group II and III mGluRs predominantly couple to Gα_i/o_ proteins to inhibit adenylyl cyclase, reduce cAMP levels and thereby reduce presynaptic glutamate release [2, 12, 14]. Thus, both receptor groups can play a key role in modulating synaptic plasticity and long-term depression. Furthermore, Group II and III mGluRs can regulate other cell signaling pathways required for synaptic transmission and plasticity independent of G proteins, such as PI3K/Akt and ERK1/2 pathways [32-34]. Taken together, the ubiquitous expression profile and ability to activate a variety of cell signaling mechanisms in the brain implicates mGluRs in many neurological disorders, including AD [8, 13, 15, 16, 19, 35, 36].

## 3 ALZHEIMER’S DISEASE

Alzheimer’s disease is a progressive age-related neurodegenerative disease that presents in elderly patients with memory loss, cognitive decline, and neuropsychiatric manifestations. AD is the most common form of dementia and represents 60-70% of dementia cases [37]. It is estimated that there are approximately 44 million people worldwide living with AD and this number is expected to at least double by the year 2050 [38]. AD disproportionately affects women who account for more than 60% of AD cases [38]. Two major pathological hallmarks of AD have been well-described in the literature that include extracellular plaques formed from insoluble β-amyloid (Aβ) peptide oligomers and the hyperphosphorylated microtubule assembly and stabilization protein, Tau [37]. Additionally, excessive production of mitochondrial reactive oxygen species (ROS) and/or reduced function of endogenous antioxidant systems has been reported in various neurodegenerative diseases, including AD. The oxidative damage to membrane lipids, proteins, and DNA leads to detrimental structural and functional changes in neurons and evidence suggests that oxidative stress can be an early and pervasive indicator of AD [39-41]

Aβ is a normal sequential proteolytic cleavage product of the membrane-anchored glycoprotein, amyloid precursor protein (APP) by β and ɣ-secretases and is normally found in the healthy brain as a soluble protein at low picomolar levels. At low levels, these peptides do not contribute to neurodegeneration, but when concentrations of the Aβ42 peptide become elevated, they oligomerize to form neurotoxic signaling complexes in AD brain [42]. Pathological overproduction of Aβ42 triggers its aggregation into soluble oligomers that may be the primary neurotoxic toxic species that also facilitate the hyper-tau phosphorylation and further contributes to AD progression [43, 44]. Aβ42 oligomers mediate their neurotoxicity by triggering membrane depolarization and excessive Ca^2+^ influx, impairing mitochondrial function, activating microglia leading to robust neuroinflammation and increasing the production of reactive oxygen species [13, 44, 45].

Tau is a microtubule associated protein found in neuronal axons and plays a key role in maintaining the stability of microtubules. Pathological hyperphosphorylation of tau triggers its aggregation, impairs its ability to support axonal transport of cellular cargoes, and its ability to regulate chromatin structure unfolding, resulting in altered gene transcription and an overall reduction in synaptic excitability [46-48]. In this review, we will focus on how Aβ and tau impair the signaling of mGluRs in neuroglia and how this contributes to the pathophysiology of AD.

## 4 NEUROGLIA

Neuroglia or glial cells are non-neuronal cells in the nervous system that were believed to represent the cytological majority of cells found in the CNS, a claim that remains controversial based on the current evidence [49]. While neuroglia are less electrically excitable than neurons and do not form classical functional synapses, they protect neurons and provide the metabolic and structural support required for synaptic function [50]. In the brain, neuroglia are composed of astrocytes, microglia, and oligodendrocytes. They are fundamental for brain homeostasis and neuroglial dysfunction has been found to contribute to many brain pathologies, including AD [50]. There is an increasing body of experimental evidence suggesting that aberrant signaling of mGluRs in neuroglial contributes to impaired AD brain function [50-52]. We will attempt to outline the contribution of the major mGluR subtype(s) in each cell type to AD pathophysiology in the coming sections of this review.

### 4.1 Astrocytes

Astrocytes are the most prevalent of glial cell types and they have numerous processes emanating from their cell bodies, giving them a star-like shape and hence, their name. Astrocytes surround the neurons and provide structural and metabolic support for neurons. They also release growth factors and regulate the uptake and release of many neurotransmitters, including glutamate. Moreover, astrocytes liaise the neurons to capillaries to regulate neurovascular coupling and cerebral blood flow, and therefore play a major role in maintaining the blood brain barrier permeability reviewed in [53-56]. Interestingly, astrocytes are responsible for over 90% of glutamate reuptake from the synaptic cleft *via* the glutamate transporter-1 (GLT1). Following uptake, glutamate is converted mainly into glutamine by the glutamine synthetase inside astrocytes and then transported back to the presynaptic neuron where glutamate is resynthesized and packaged into synaptic vesicles. This glutamate-glutamine shuttle is the key for glutamatergic signaling, and thereby for learning and memory [57, 58].

#### 4.1.1 mGluR Expression in Astrocytes

Expression of mGluR5 was detected in hippocampal and cortical astrocytes, but the expression of the two splice variants mGluR5a and mGluR5b appears to decline during development albeit to a greater extent in the case of the “b” splice variant [59]. On the other hand, mGluR1 shows a limited expression profile and is only detected in a small proportion of astrocytes derived from the spinal cord [60]. mGluR3 is significantly expressed in the cortical and hippocampal astrocytes and is present at all developmental stages and is considered the most abundant mGluR subtype in astrocytes, while mGluR2 expression was not detected [61, 62]. mGluR4 expression in astrocytes is debated; some studies have detected the receptor in cortical astrocytes while others have not [51]. No robust expression of mGluR6, 7 or 8 expression is detected in astrocytes under normal physiological conditions [62-64]. Thus, it is evident that mGluR3 and mGluR5 are the two most abundant mGluRs in astrocytes.

#### 4.1.2 Aβ Oligomer Neurotoxicity and Astroglial mGluRs

Reactive astrocytes have been detected around the Aβ plaques in AD mouse models and patients [65, 66]. Moreover, a robust increase in mGluR5 expression is detected in astrocytes surrounding Aβ plaques in both mouse models and patients with AD [67-70]. Interestingly, Aβ42 oligomers are capable of forming a ternary complex with mGluR5 and cellular prion protein (PrP^C^) to trigger receptor clustering and the pathological signaling of the receptor in neurons [22, 30, 71]. Additionally, Aβ42 oligomers rapidly bind mGluR5 on the plasma membrane of astrocytes and induce receptor clustering and Ca^2+^ oscillations [68]. Moreover, the pharmacological and genetic ablation of mGluR5 in mouse models of AD mitigated the cognitive impairment, Aβ42-related pathology and astrogliosis [28-30, 72]. Thus, it is evident that aberrant mGluR5 signaling in astrocytes contributes to the pathophysiology of AD. It is important to note that mGluR5 activation in cultured astrocytes can also provide a neuroprotective role by increasing the astrocytic release of brain derived neurotrophic factor (BDNF) to support neuronal survival, but it remains unclear whether astroglial mGluR5-dependent neuroprotection is evident *in vivo* and whether it is abolished by Aβ42 [73].

Since intracellular Ca^2+^ signalling in astrocytes is the key for communication between neurons and astrocytes [57], disruption of astroglial Ca^2+^ homeostasis and Ca^2+^-dependent signaling may play a major role in AD pathogenesis [74]. Transcriptomic analysis of astrocytes from AD patient reveals a disruption in the expression of numerous gene transcripts, including 32 genes transcripts associated with Ca^2+^ signaling, indicating an obligatory contribution of altered astrocyte Ca^2+^ dynamics to AD pathogenesis [75]. Indeed, Aβ42 oligomers disrupt Ca^2+^ homeostasis in hippocampal astrocytes in an mGluR5-dependent mechanism. Specifically, Aβ42 oligomers activate calcineurin (CaN) that can then trigger the nuclear translocation of the transcription factor Nuclear Factor-κB (NF-κB) to upregulate both mGluR5 and IP_3_R1, leading to disruption in Ca^2+^ handling within astrocytes and impairment in synaptic transmission [76, 77]. Aβ42 oligomers also directly activate CaN to dephosphorylate mGluR5 resulting in reduced mGluR5 desensitization and reduction in receptor turnover, this may explain the enhanced receptor expression in astrocytes around Aβ plaques [78]. Additionally, activation of NF-κB can be associated with robust expression of proinflammatory markers in astrocytes that further exacerbate AD pathology [79] and indeed astrocytic mGluR5 blockade prevents the secretion of the inflammatory cytokines Interleukin 6 and 8 by astrocytes [80]. Thus, inhibiting mGluR5 can alleviate the neurotoxic burden of astrocytes in AD brain that may be a contributor to the disease-modifying outcomes reported in AD mice following mGluR5 silencing [28, 30, 72].

In contrast, astroglial mGluR3 may play a protective role against Aβ42 neurotoxicity, and a reduction in mGluR3 activity in astrocytes is reported in AD [56, 81, 82]. Specifically, activation of mGluR3 in astrocytes promotes the non-amyloidogenic cleavage of APP by inhibiting β-secretase 1 (BACE1) expression and enhancing the expression of the α-secretase ADAM10 and 17, leading to increased release of the neuroprotective soluble APPα (sAPPα) [81]. sAPPα acts in a paracrine and autocrine manner to stimulate Aβ42 oligomer uptake by astrocytes and microglia [83, 84]. Additionally, mGluR3 activation also enhances the expression of BDNF that may play a key role in preventing Aβ42 oligomer-mediated neurotoxicity and promoting the survival and differentiation of neurons [84, 85]. Moreover, pharmacological activation of mGluR3 in astrocytes protects neurons against Aβ42 oligomer-induced neurotoxicity by enhancing the production of transforming growth factor β1 (TGF-β1) and the paracrine activation of the anti-apoptotic and anti inflammatory mechanisms in neurons [56, 86]. It is worth noting that TGF-β1 plays a key role in synaptic plasticity and memory formation and impaired TGF-β1 signaling is reported to contribute to neuroinflammation and cognitive decline in AD [87]. Together, it is evident that mGluR3 activators can be a promising approach to enhance the disease-modifying properties of astrocytes in the AD brain.

**Fig. (1):**
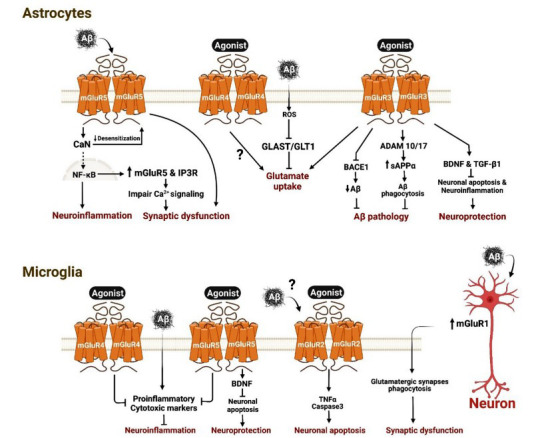
β-Amyloid (Aβ) neurotoxicity and neuroglial mGluRs. **In astrocytes:** β-Amyloid (Aβ) binds to mGluR5 and activates calcineurin (CaN) that facilitate the nuclear translocation of nuclear factor κB (NF-κB) and enhances the expression of neuroinflammatory markers. NF-κB increases expression of mGluR5 and inositol trisphosphate receptor 1 (IP3R1), leading to impaired Ca^2+^ signaling and synaptic dysfunction. CaN can also reduce mGluR5 desensitisation that contributes to synaptic dysfunction. Aβ increases the production of reactive oxygen species (ROS) that inhibits glutamate-aspartate transporter (GLAST) and Glutamate transporter 1 (GLT1), leading to a reduction in glutamate uptake. Agonist-dependent activation of mGluR3 and mGluR4 can enhance glutamate uptake, yet it remains debateable in the case of mGluR4. Agonist-dependent activation of mGluR3 inhibits β-secretase 1 (BACE1) that reduces the production of Aβ and activates α-secretases ADAM10 and 17 that increases the production of soluble amyloid precursor protein α (sAPPα), resulting in the phagocytosis of Aβ by astrocytes and microglia. Agonist-dependent activation of mGluR3 can also increase the production of brain derived neurotrophic factor (BDNF) and transforming growth factor β1 (TGF-β1) that reduces neuronal apoptosis and neuroinflammation and contribute to overall neuroprotection. **In microglia:** Aβ triggers the release of many proinflammatory and cytotoxic markers leading to neuroinflammation and neurotoxicity. Agonist-dependent activation of mGluR4 and mGluR5 reduces neuroinflammation. mGluR5 activation enhances the production of BDNF that supports neuroprotection by reducing apoptosis. Agonist or possibly Aβ-dependent activation of mGluR2 enhances the release of tissue necrosis factor α (TNFα) and activates caspase3 leading to neuronal apoptosis. Aβ increases the expression of mGluR1 in neurons that can act in paracrine manner to trigger the phagocytosis of glutamatergic synapses by microglia and contribute to synaptic dysfunction.

Aβ42 oligomers are also known to reduce astroglial glutamate uptake capacity that is explained by an oxidative stress-induced reduction in the function of glutamate-aspartate transporter (GLAST) and GLT-1 [88]. Interestingly, activation of Group II and III mGluRs in astrocytes can be partially neuroprotective by enhancing glutamate uptake and inhibiting postsynaptic glutamate signaling [89]. However, in one study, mGluR4 activation was found to downregulate GLT-1, and therefore, it is evident that the role of Group III mGluRs in astrocytes remains controversial [90]. Therefore, delineating the precise physiological function of mGluR4 in astrocytes and how this function is disrupted by Aβ can be very useful in designing novel therapies for AD and possibly other neurological conditions characterized by excessive glutamate release (Fig. **1**).

### 4.2 Microglia

Microglia are the resident macrophages of the brain and form the first and critical line of active immune defense and they account for up to 16% of the total cell population in certain brain regions [91]. The plasticity of the microglia allows them to undergo various structural changes in response to stimuli. In a “resting” state, ramified microglia have a small static cell body with long branching processes that are constantly surveying its surrounding for threats and are capable of transforming into the “reactive” form. Upon reactivation, microglia transform into antigen presenting, cytotoxic, and inflammation-mediating non-phagocytic microglia, capable of undergoing rapid proliferation, and then progress to become large ameboid phagocytic microglia which are the quintessence of immune-response. The phagocytic microglia can travel to the site of injury, engulf foreign and harmful material, and stimulate pro-inflammatory signaling, amplifying the immune response reviewed in [92, 93].

#### 4.2.1 mGluR Expression in Microglia

In cultured microglia, the expression of mGluR1 is minor to negligible, whereas, mGluR5 is heavily expressed [51]. mRNA and receptor protein expression of the two members of group II mGluRs (mGluR2 and mGluR3) are also detected [94, 95]. Cultured microglia also express mRNA and receptor protein for most members of group III mGluRs, including mGluR4, mGluR6, and mGluR8. However, the expression of mGluR7 has not been detected in microglia [96].

#### 4.2.2 Aβ Oligomer Neurotoxicity and Microglial mGluRs

Microglia play a key role in CNS immunostasis but, reactive microgliosis (an increase in the number of microglia) is recognized as a hallmark of many CNS pathologies, including AD [91]. Moreover, mGluRs have been shown to be involved in modulating microglial function in many brain pathologies. For instance, genetic ablation of mGluR5 in BACHD model of Huntington’s disease enhances the cortical microglial population whereas pharmacological activation of mGluR2/3 in zQ175 mouse model of Huntington’s disease reduces the striatal microglia numbers [34, 97]. Additionally, α-synuclein-mediated microgliosis, a key driver of Parkinson’s disease pathogenesis, has been shown to be tampered by a selective mGluR5 agonist [98]. More so, activation of mGluR5 in rodent models of traumatic brain injury leads to an inhibition of microglia activation and reduction in pro-inflammatory cytokines [99, 100]. Interestingly, genetic or pharmacological silencing of mGluR5 reduced motor neuronal death and microglia activation in the SOD1G93A mouse model of amyotrophic lateral sclerosis [101, 102].

Reactive microglia are detected in the vicinity of Aβ plaques and hyperphosphorylated tau tangles [103, 104]. The phagocytic function of activated microglia against Aβ is well established, but the degradation of Aβ after phagocytosis is slow and may not be effective in clearing the Aβ burden [105]. The detrimental neurotoxic effect of microgliosis in AD is primarily mediated by the proinflammatory cytokines/chemokines secreted by microglia [106, 107]. Additionally, Aβ42 oligomers can activate the complement cascade and drive synapse elimination by activated microglia [108]. Aβ42 oligomers also promote the release of glutamate from microglia that can dramatically elevate neuronal Ca^2+^ levels and contribute to neuronal death [109].

While it is evident that astroglial mGluR5 contributes to Aβ42 oligomer-mediated neurotoxicity, mounting evidence suggests that microglial mGluR5 offers some neuroprotective function. Studies from the Faden group demonstrated that agonist-dependent activation of mGluR5 inhibited microglial associated inflammation and neurotoxicity *via* Gα_q/11_/PLCβ-dependent mechanism [110, 111]. Moreover, intrahippocampal injection of Aβ42 oligomers in mice is associated with microgliosis that is mitigated by treatment with an mGluR5 positive allosteric modulator [112]. mGluR5 activation in microglia also enhances the production of BDNF, protects from apoptosis, and reduces the accumulation of reactive oxygen species and inflammatory mediators [113]. Additionally, antagonism of mGluR5 in microglia increases endoplasmic reticulum stress and mitochondrial dysfunction and drives microglia towards a pro-inflammatory state [114]. On a different note, the activation of microglia recruits astrocytes and amplifies astrocytic glutamate release and potentially mGluR5-mediated neurotoxicity, but it is not clear whether this observation is evident in the AD brain [115, 116]. Thus, it is imperative to study in the future the basis for the opposing mGluR5 function between astrocytes and microglia and the relative contributions of mGluR5 signaling in each cell type to AD pathophysiology at different stages of the disease process. Such findings will be critical in guiding the selection of the most effective mGluR5 ligand to halt the progression of AD, at the appropriate stage. However, it is important to note that mGluR5 inhibition provides exquisite disease-modifying outcomes in AD brain and this may suggest that Aβ42-triggered pathology is more robust in astrocytes compared to microglia [29, 72]. While the expression of mGluR1 is negligible in microglia, accumulation of Aβ fibrils results in the upregulation of mGluR1 in rat hippocampal neurons injected with Aβ fibrils. This upregulation in mGluR1 downstream signaling triggers paracrine activation of microglial phagocytosis and elimination of glutamatergic synapses that contributes to the synaptic dysfunction and memory deficits observed in AD [117].

Unlike astrocytes, agonist- or Aβ (Aβ_25-35_)-dependent activation of group II mGluRs is associated with microglia-mediated neurotoxicity and induces microglial apoptosis [94]. Agonist-dependent neurotoxicity is found to be due to preferential activation of microglial mGluR2, but not mGluR3, which induces TNFα release and caspase-3 activation [118]. Interestingly, this was not the only occasion where mGluR2 and mGluR3 exhibited differential effects on microglial function. Specifically, myelin-evoked neurotoxicity in cultured rat microglia is exacerbated by microglial mGluR2 activation but is abrogated by selective mGluR3 activation [119]. This neuroprotective response of mGluR3 activation may be attributable to the release of TGF-β1, but this hypothesis has not been tested [87]. The molecular basis for such a differential response between the activation of two members of the group II mGluR family remains elusive. However, it suggests that selective activators (agonists or positive allosteric modulators) of mGluR3 but not the non-selective mGluR2/3 activators are a viable strategy to reverse Aβ-triggered pathology in both astrocytes and microglia and slow AD progression.

The activation of group III mGluRs with a non-selective agonist protects neurons against microglia-mediated neurotoxicity [96]. Additionally, selective activation of mGluR4 in primary mouse microglia reduces pro-inflammatory response, which is not evident in microglia derived from mGluR4 KO mice [120]. This suggests that activators of group III mGluRs and specifically mGluR4 may have disease-modifying effects in AD and other brain disorders with inflammatory insults. It remains less clear how activation of group III mGluR prevents microglial activation, but a reduction in glutamate release is surely one of the proposed hypotheses (Fig. **1**) [96].

### 4.3 Oligodendrocytes

Oligodendrocyte progenitor cells (OPCs) differentiate to form mature oligodendrocytes that can extend multiple processes to encase nearby axons with myelin. This myelin sheath not only supports and insulates axons in the CNS, but also induces the clustering of sodium channels at the node of Ranvier that is important for saltatory conduction of action potential. Oligodendrocytes also provide metabolic support to the neurons and produce many neurotrophic factors reviewed in previous studies [121, 122].

#### 4.3.1 mGluRs Expression in Oligodendrocytes

mGluR1/5 and mGluR2/3 are expressed in oligodendrocytes, but their expression is developmentally regulated and generally presents with high expression levels in early stages followed by downregulation at maturity [56, 123-125]. mGluR4, 7 and 8 are expressed in both OPCs and oligodendrocytes, whereas mGluR6 is found to be expressed in oligodendrocytes only [13, 62].

#### 4.3.2 Aβ42 Oligomer Neurotoxicity in Oligodendrocytes

Disruption in the oligodendrocyte function can be associated with detrimental outcomes to neuronal conduction and can lead to axonal degeneration, as reported in AD [126]. Specifically, Aβ oligomers can disrupt oligodendrocyte differentiation and function, and Aβ42 oligomer-induced oxidative stress can drive oligodendrocyte apoptosis [127-129]. Aβ42 oligomers also impair Ca^2+^ homeostasis and increase oxidative stress burden in oligodendrocytes leading to their death, axonal dysfunction, and cognitive impairments in mice [130]. Moreover, hyperphosphorylation of tau can impair its ability to stabilize microtubules and thereby disrupt the myelination function of oligodendrocytes [126]. However, the role of mGluRs in meditating Aβ42 oligomer and/or tau-induced toxicity in oligodendrocytes remains largely unknown. Interestingly, activation of NF-κB has been detected in oligodendrocytes after exposure to Aβ42 oligomers and since NF-κB is regulated by group I mGluRs [129, 131, 132], it is possible that mGluR1/5 plays a role in oligodendrocyte dysfunction in AD. In contrast, activation of group I mGluRs is found to enhance the survival of OPCs by reducing oxidative stress, a key mechanism of Aβ42 oligomers-induced neurotoxicity [44, 123]. Additionally, mGluR4 activation enhances the differentiation and branching of oligodendrocytes under conditions of excitotoxicity [133]. Thus, along with its ability to reverse microglia-induced inflammation [120], mGluR4 activators could be a promising approach to restore the axon regenerative capacity of oligodendrocytes in AD. We trust that a special focus on the effects of Aβ42 oligomer on oligodendrocytic mGluRs is required as it may shed light on new pharmacological strategies that can reverse or slow axonal degeneration in many neurological disorders.

### 4.4 Tau and Neuroglial mGluRs

The mechanism by which hyperphosphorylated tau protein contributes to neuroglial dysfunction is not well understood. However, it is evident that the function of most neuroglia is dependent on their ability to interact with neurons through their processes [50]. Since tau protein is the key to the stabilization of microtubules and consequently the support of axonal transport of cellular cargos, it is conceivable that the impaired function of tau protein in AD will impair neuroglial processes formation and function [47, 48]. Additionally, evidence suggests that tau oligomers trigger synaptic dysfunction by binding to PrP^C^, which is also known to be a part of the neurotoxic mGluR5/Aβ42 oligomer/ PrP^C^ signaling complex [22, 30, 134]. Therefore, it is likely that mGluRs play a pivotal role in mediating neuroglial dysfunction in tauopathies and this role requires further extensive investigation.

## CONCLUSION

The precise role of each mGluR subtype in neuroglial function requires further investigation, but it is evident from the current studies that neuroglial mGluRs play a key role in supporting synaptic plasticity and can contribute to AD pathophysiology. In astrocytes, evidence indicates that mGluR5 contributes to the neurotoxic effects of Aβ42, whereas activation of mGluR3 supports a neuroprotective function. In microglia, evidence shows that activation of mGluR5 and mGluR3 can be protective against Aβ42-induced neuroinflammation while the activation of mGluR2 can exacerbate neuroinflammation. It is also evident that the activation of mGluR4 in astrocytes, microglia and oligodendrocytes can reverse some of Aβ42-mediated neurotoxic mechanisms. More so, mGluRs are important in supporting the myelinating function of oligodendrocytes and should be carefully investigated as potential targets to reverse axonal degeneration disorders.

## FUTURE DIRECTIONS

While it is evident that neuroglial mGluRs play a key role in cognitive function, many fundamental questions about their precise contribution to AD pathophysiology remain unanswered. Thus, it will be important in the future to examine whether Aβ and tau can directly interact with the various neuroglial mGluR similar to what was reported for mGluR5 in neurons [30, 71]. It will also be crucial to delineate the pathological mechanism(s) triggered by such interactions and how they can alter the physiology of the different neuroglial cell types. Moreover, conditional deletion of each major mGluR subtype from the three neuroglial cell types in mouse models of AD will provide *in vivo* insights into their precise contribution to AD pathophysiology at different stages of the disease. Furthermore, evidence indicates that at least three members of the mGluR family, mGluR2/3 and 5, exhibit sex-biased signaling in neurons [30, 34] and, therefore, it is imperative that we explore whether this sex-selective mGluR signaling is also evident in neuroglia and whether it translates into differences in the pathophysiology of AD between both sexes. As discussed earlier, mGluR5 and mGluR2/3 can exhibit either neurotoxic or neuroprotective functions depending on the neuroglial cell type. Therefore, understanding the overall contribution of each neuroglial mGluR subtype to AD pathogenesis will help guide the selection of the most effective pharmacological approach for the treatment of AD. This is of a particular interest for mGluRs because of the abundance of many readily available agonists, antagonists and allosteric modulators that offer high selectivity and versatile pharmacokinetic profile suitable for therapeutic application.
